# Clinical Outcomes of Soft Tissue Preservation Surgery With Hydroxyapatite-Coated Abutments Compared to Traditional Percutaneous Bone Conduction Hearing Implant Surgery—A Pragmatic Multi-Center Randomized Controlled Trial

**DOI:** 10.3389/fsurg.2020.00005

**Published:** 2020-03-05

**Authors:** M. van Hoof, S. Wigren, J. Ivarsson Blechert, M. A. Joore, D. J. M. Mateijsen, S. J. H. Bom, J. Stalfors, Måns Eeg-Olofsson, O. Deguine, A. J. M. van der Rijt, M. C. Flynn, J. Marco Algarra, R. J. Stokroos, The Angelfish Collaborative

**Affiliations:** ^1^School for Mental Health and Neuroscience (MHENS), Ear, Nose and Throat (ENT) Department, Maastricht University Medical Center, Maastricht, Netherlands; ^2^Cochlear Bone Anchored Solutions AB, Mölnlycke, Sweden; ^3^Department of Clinical Epidemiology and Medical Technology Assessment, Maastricht University Medical Center, Maastricht, Netherlands; ^4^ENT Department, Catharina Hospital, Eindhoven, Netherlands; ^5^ENT Department, Deventer Hospital, Deventer, Netherlands; ^6^Department of Otorhinolaryngology, Sahlgrenska University Hospital, and Institute of Clinical Sciences, Sahlgrenska Academy at the University of Gothenburg, Gothenburg, Sweden; ^7^ENT Department, Purpan Hospital, Toulouse, France; ^8^ENT Department, Amphia Hospital, Breda, Netherlands; ^9^University of Newcastle, Callaghan, NSW, Australia; ^10^ENT Department, Clinical University Hospital, Valencia, Spain; ^11^Department of Otolaryngology, Head and Neck Surgery, Brain Center Rudolph Magnus, University Medical Center Utrecht, Utrecht, Netherlands

**Keywords:** BAHA, RCT - randomized controlled trial, hydroxyapatite, soft tissue preservation, surgery

## Abstract

**Background:** Soft tissue preservation using a hydroxyapatite-coated abutment in bone conduction hearing implant surgery may lead to improved clinical outcomes over the short (1 year) and long term (3 years).

**Methods:** In this open multi-center, randomized (1:1), controlled clinical trial, subjects with conductive, mixed hearing loss or single-sided sensorineural deafness were randomly assigned to receive the conventional intervention, a titanium abutment with soft tissue reduction surgery (control), or a new intervention, a hydroxyapatite-coated abutment with soft tissue preservation surgery (test). The primary efficacy outcome was the combined endpoint of numbness, pain, peri-abutment dermatitis, and soft tissue thickening/overgrowth after 1 and 3 years.

**Results:** The Intention-to-treat (ITT) population consisted of 52 control subjects and 51 test subjects. The difference between the groups after 1 year of follow-up as measured by the primary efficacy outcome was not statistically significant (*p* = 0.12) in the ITT population (*n* = 103), but did reach statistical significance (*p* = 0.03) in the per-protocol (PP) population (*n* = 96). It showed an advantage for the test group, with over twice as many subjects (29%) without these medical events during the first year compared to the control group (13%). After 3 years, the difference between the two groups had declined and did not reach statistical significance (24 vs. 10%, ITT *p* = 0.45). Secondary outcome measures which showed a statistical significant difference during the first year, such as surgical time (15 vs. 25 minutes, *p* < 0.0001), numbness (90 vs. 69% of subjects experienced no numbness at 1 year, *p* < 0.01), neuropathic pain at 3 months (*p* = 0.0087) and the overall opinion of the esthetic outcome (observer POSAS scale at 3 months, *p* < 0.01) were favorable for the test group. More soft tissue thickening/overgrowth was observed at 3 weeks for the test group (*p* = 0.016). Similar results were achieved for the long term follow up.

**Conclusions:** Soft tissue preservation with a hydroxyapatite-coated abutment leads to a reduction in the combined occurrence of complications over the first year which is not statistically significant in the ITT population but is in the PP population. This effect decreased for the long-term study follow up of 3 years and did also not reach statistical significance.

## Introduction

### The Bone Anchored Hearing Aid

**Figure 1:**
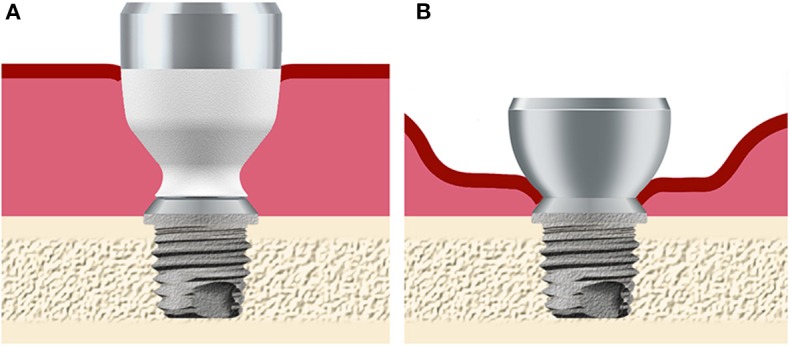
Overview of the abutment design and soft tissue status after surgery. **(A)** The hydroxyapatite-coated titanium abutment is placed in full thickness skin in the *test group*. **(B)** The all-titanium abutment is placed in skin where subdermal tissue has been removed using soft tissue reduction in the *control group*. Both abutments connect to the same implant fixture, which is placed in the skull bone behind the ear.

Hearing impairment poses a significant global burden (1) and has been linked to cognitive decline, depression and a number of disabilities (2). For certain patients with conductive or mixed hearing losses, current surgical interventions and/or conventional hearing aids may not provide adequate hearing rehabilitation (3). The Bone Anchored Hearing Aid has been developed as an alternative treatment in these cases (4). The system under investigation consists of a screw-shaped titanium implant (5), which is fixed into the skull and osseointegrates with the surrounding bone (6). A percutaneous titanium abutment (Figure 1) then connects the implant to the sound processor, which provides amplification to the patient. Based upon the principle of bone conduction (7), this system is capable of bypassing the defective outer or middle ear when transmitting external sound to the cochlea. The system is also indicated for patients with single-sided sensorineural deafness (8), where bone-conducted vibrations are routed from the impaired side to the unaffected contralateral cochlea (9). This allows for the restoration of some of the advantages of having two functional ears; most importantly, it reduces the *head shadow effect* (10).

### Surgical Techniques and Clinical Outcomes

Significant effort has been taken to optimize the surgical procedure surrounding BAHA implantation (11) and high implant survival rates are achievable (12) with modern surgical procedures. However, during older procedures, extensive removal of the subcutaneous tissue around the abutment (*soft tissue reduction*) was advocated in order to reduce soft tissue mobility (13, 14) and to prevent the formation of skin pockets in which bacteria could accumulate and cause infection. Soft tissue reduction surgery became the gold standard (15, 16), even though it may result in localized hair loss, scarring, visual indentation in the skin and loss of sensibility in the area surrounding the abutment (17, 18). These complications are likely related to the fact that dermal tissues are severely undermined, hair follicles are often permanently removed, and the nerve innervation is severed, hampering normal skin defense mechanisms. Later, a vertical incision with tissue thinning was introduced and has been used during implantation in over 140,000 patients globally, but cosmetic and clinical drawbacks remain to be addressed further (19). Research addressing soft tissue complications using this method have been performed for percutaneous implants used in adjacent fields (20) and also in the bone conduction hearing implant literature (21–23). Complications such as pain, numbness and cosmetic issues were infrequently reported before the onset of this trial. These complications can be a burden for patients and decrease the perceived benefit of the system and also lead to considerable medical costs (24). Reducing cosmetic issues associated with the intervention may increase the acceptance of by patients and the general public (25). For instance, retained hair can function as a way of concealing the abutment and the sound processor (26).

### Hypothesis

The clinical trial aims to investigate long-term soft tissue outcomes using percutaneous implants that do not require soft tissue thinning. Surgically undermining adjacent tissues may interfere with healing and integration between the soft tissue and the abutment is important for favorable healing outcomes (27, 28). Sealing the abutment-skin interface could reduce the impact of bacterial infiltration (29) which thrive in biofilms (30) in niches created between the abutment and surrounding skin. Viable device-tissue adherence *(“integration”*) could provide the immune system access to the abutment surface to control this interface (31, 32). Integration does not occur with standard titanium abutments (33) and the use of an abutment surface allowing soft tissue integration was thought necessary for good clinical results. An abutment coating made of hydroxyapatite (HA), a naturally occurring compound present in the bony tissue and in teeth (which can maintain life-long soft tissue penetration), was chosen for this purpose (Figure 1A). As previous investigations have suggested, soft tissue-abutment integration is possible in animals (34, 35). Recently, it has been shown to be clinically possible in humans (32, 36) using this HA-coated abutment. It was hypothesized that the combination of skin preservation and tissue integration could improve the subjective benefit of the treatment, by improving the cosmetic aspects and reducing post-operative complications without compromising long-term clinical outcomes.

### Objectives

This multi-center randomized controlled clinical trial aims to investigate post-operative outcomes following implantation of a HA-coated abutment with soft tissue preservation during surgery. Outcomes will be compared against implantation of a traditional titanium abutment with soft tissue reduction during surgery. Complications and cosmetic outcomes will be monitored over the first-year and up to the third-year post implantation for each treatment arm.

## Methods

### Ethics

The final protocol, consent documentation and substantial amendments were approved by the respective ethics committees at each site (De Medisch Ethische Toetsingscommissie, Maastricht, the Netherlands; Regionala etikprövningsnämnden, Göteborg, Sweden; Del Comite Ético de Investigatión Clinica del Hospital Clinico Universitario de Valencia, Spain; Comité de Protection des Personnes Sud-Ouest et Outre-Mer I/ Agence nationale de sécurité du medicament et des produits de santé, France). The ethics committee in the Netherlands (Maastricht) approved this study for the other Dutch centers who participated. The board of directors of these hospitals (Amphia Hospital, Breda; Catharina Hospital, Eindhoven; Deventer Hospital, Deventer; the Netherlands) subsequently approved conducting this clinical trial according to local legislation. The study was conducted in compliance with the provisions of the Declaration of Helsinki and ISO 14155:2011 “Clinical investigation of medical devices for human subjects—Good clinical practice.” All subjects provided written informed consent. The study was registered on ClinicalTrials.gov (NCT01796236).

### Study Design

The trial was designed by the sponsor to be an international, multi-center, open, prospective, randomized controlled trial in conjunction with the authors. The present paper reports the results from the analysis of clinical data performed after 1 year as well as long-term clinical data collected up to 3 years of follow-up. Direct medical resource use and costs, patient reported outcomes and implant stability measurements are also analyzed as part of the trial, but will be presented in separate publications. Subjects were randomly assigned before surgery, in a 1:1 ratio, to one of the study treatment arms without subject variable stratification. Randomization was performed with site stratified permuted blocks with a variable block size and the use of an automated Web-based system (dSharp, Göteborg, Sweden). Data collection and study visits followed the flowchart in Supplemental Figure 1. No major changes were made to the study protocol or outcome measures during the trial.

### Study Treatment

The test device was the HA-coated Cochlear™ Baha® BA400 Abutment (length 6, 8, 10, or 12 mm) placed using soft tissue preservation surgery. The control device was the all-titanium Cochlear Baha BA300 Abutment (length 6 or 9 mm). Both abutment types (Figure 1) were connected to a Cochlear Baha BI300 Implant. All devices are manufactured by Cochlear Bone Anchored Solutions AB (Mölnlycke, Sweden). An appropriately sized abutment was chosen according to the surgical manual as provided by Cochlear. In the test group, the surgery consisted of a linear incision *without* soft tissue reduction (37) followed by the placement of the implant with pre-mounted abutment through a 5 mm punched hole approximately 10 mm lateral from the initial linear incision. In the control group, surgery was performed using a linear incision and soft tissue reduction (38). After the surgical procedure, a healing cap with dressing was used during the initial healing phase in both groups. Attachment of the sound processor to the abutment was advocated from 3 weeks post-surgery.

### Subject Selection

Adult subjects (≥18 years) with a conductive or mixed hearing loss or single-sided sensorineural deafness who were eligible for a bone conduction hearing implant system were prospectively enrolled in the study between 25-01-2013 and 19-05-2014. The trial was conducted in both academic (Clinical University Hospital, Valencia, Spain; Purpan Hospital, Toulouse, France; Maastricht University Medical Center, Maastricht, the Netherlands; Sahlgrenska University Hospital, Göteborg, Sweden) and non-academic hospitals (Amphia Hospital, Breda; Catharina Hospital, Eindhoven; Deventer Hospital, Deventer; the Netherlands) across Europe to reflect the conventional availability of the intervention. Exclusion criteria were bilateral implantation, uncontrolled diabetes, conditions that could jeopardize osseointegration and/or wound healing, inability to follow the cleaning instructions or to complete study-related questionnaires, concurrent participation in another clinical investigation, insufficient bone quality/quantity as observed during surgery or a condition that may have a substantial impact on the outcome of the investigation as judged by the investigator.

## Outcome Measures

Baseline characteristics were collected at the pre-operative visit and included potential risk factors, such as smoking. At surgery, skin thickness was measured using a needle and a ruler, prior to injecting local anesthesia. Surgery time, defined as the time between the first incision and the last suture, was recorded. Wound healing was evaluated during the first 6 months. Different aspects of the peri-abutment skin and soft tissue were evaluated at each visit post-surgery. The Holgers index (39) was used to classify any presence of peri-abutment dermatitis (i.e., skin inflammation), and was recorded at every post-surgical visit and at any extra visits due to dermatitis-related adverse events. Soft tissue thickening and overgrowth was assessed using a newly developed scale (“*Soft tissue thickening/overgrowth*,” Table 1). Visible abutment length was measured using a standardized ruler. Pain was measured in two separate dimensions: *neuropathic pain*, characterized as sharp or radiating pain not necessarily associated with the area surrounding the implant, and *direct pain*, relating to the scar and area around the implant. Both dimensions were measured on a scale from 1 to 10, where 1 represented no pain and 10 represented the worst imaginable pain. Numbness (sensibility loss) was assessed using a newly developed scale *(“Numbness scale*,” Table 1). Any abutment changes were recorded. The Patient and Observer Scar Assessment Scale (POSAS), previously validated for scar assessments (40), was used to assess the patient's and the investigator's (observer's) rating of the appearance of the skin surrounding the abutment. The POSAS also included a neuropathic and direct pain dimension score.

**Table 1:** Scales.

**Soft tissue thickening/overgrowth scale**	
0	No soft tissue thickening or overgrowth.
1	Slight soft tissue thickening or overgrowth.
2	Moderate soft tissue thickening or overgrowth. Local treatment and extra controls as indicated.^*^
3	Marked/distinct soft tissue thickening or overgrowth. Revision surgery is indicated.^*^
**Numbness scale**	
1	No numbness as experienced by the subject.
2	Numbness within 2 cm from the abutment as experienced by the subject.
3	Numbness within and beyond 2 cm from the abutment as experienced by the subject.

* *Should be reported as an adverse event*.

### Primary Measure of Efficacy—A Combined Outcome

The primary efficacy variable was a combined endpoint. It was calculated as the sum of four different important medical complications measured in this study over the first year and up to the third year (end of follow up): occurrence of any numbness, pain (any pain dimension score >2), peri-abutment dermatitis (*Holgers index* > 1 during standard and/or extra visits) and soft tissue thickening/overgrowth (score >1). For the combined endpoint, each medical event was counted only once per subject resulting in a score of 0 to 4 for every subject. Medical complications were also analyzed separately throughout the study.

### Safety Assessments

Adverse events and device deficiencies related or not to the intervention, were prospectively collected. Implant extrusions were recorded. Serious adverse events were reported to the ethics committees as per local requirements.

### Study Management and Oversight

Data management was performed by data managers (dSharp, Göteborg, Sweden) and the trial was monitored by a monitor (A+ Science AB, Stockholm, Sweden) contracted by the sponsor. Statistical analyses were performed by biostatisticians (Statistiska Konsultgruppen, Göteborg, Sweden) according to a pre-defined statistical analysis plan which was approved by the last author, the responsible statistician and a sponsor-representative prior to database locks (after 1 and 3 years). All primary and secondary outcomes were reported on. No *post-hoc* analyses were conducted. All data were made available to the first author for inspection and quality assurance. The first author validated the database and verified the statistical analyses. The first manuscript draft was written by the first author and edited by all co-authors. All authors support the reported analyses and subsequent interpretation of the data, which the first author validated in detail. All authors vouch for the fidelity of the study to the protocol and supported the decision to submit the manuscript for publication.

## Statistical Analysis

### Sample Size Calculation

Power calculations based on reported complication rates in the literature suggested that 50 evaluable subjects were needed in each treatment group to achieve a power of 80% with the Mantel-Haenszel two-sided chi-square test. The significance level of 0.05 was split between the two primary analyses (combined clinical outcomes endpoint of 0.0499 and direct medical cost of 0.0001 for the cost-consequence analysis).

### Analysis

For comparison between the two study groups the Mantel–Haenszel chi-square test was used for ordered categorical variables, the Mann–Whitney *U*-test for continuous variables, Fisher's exact test for dichotomous variables and the Chi-square test for non-ordered categorical variables. Pain was categorized as either *No pain* (pain score 1), *Mild* (score 2–3), *Moderate* (score 4–6) or *Severe pain* (score 7–10). All tests were two-tailed. For the primary clinical endpoint a significance level of 0.0499 was adopted (see Sample size calculation). All other tests were conducted at a 0.05 significance level. All analyses were performed using SAS® v9.4 (Cary, United States).

Efficacy analyses were performed on the intention-to-treat (ITT) population and per-protocol (PP) population. Safety analyses were performed on the Safety population, which included all surgically treated subjects. The ITT population consisted of all randomized subjects with at least one follow-up measurement post-surgery. The PP population included all subjects who completed the study according to the protocol. Subjects who were incorrectly randomized, considered major protocol violators, missed more than one study visit before the end of the first year, or terminated the study before the end of the first year were removed from the PP population. A separate analysis was performed for the primary analysis where only subjects were considered who attended every visit.

## Results

### Study Subject Demographics

**Figure 2:**
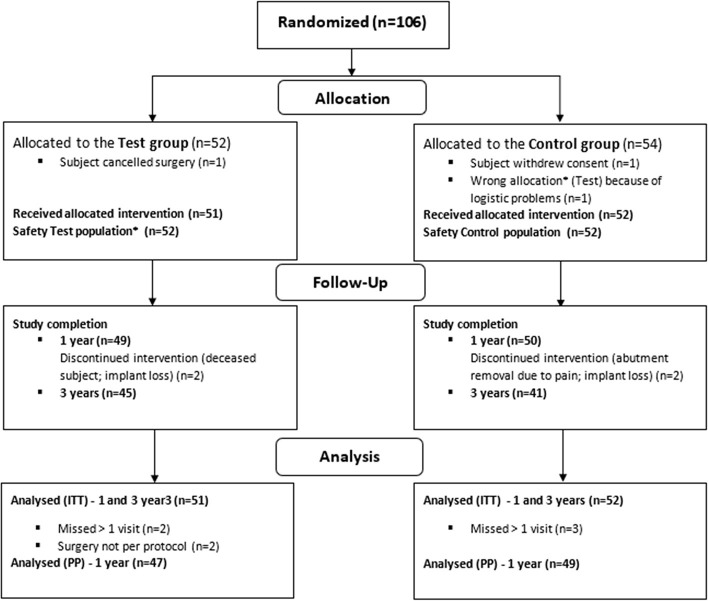
Randomization, treatment and follow-up of subjects during the study. *Due to wrong device allocation in the control group, one subject (randomized to the control group) is considered in the safety population of the test group.

One hundred six (106) subjects were enrolled in the clinical trial across the seven sites and randomized to one of the treatment groups (Figure 2). One hundred four (104) of these subjects underwent surgery and constituted the Safety population. One hundred three (103) subjects (51 test, 52 control) were included in the ITT population, and 96 subjects (47 test, 49 control) met the criteria to be included in the PP population up to the primary evaluation time point (1 year). At the end of the 3-year long-term follow up, 85% of the test group completed the study vs. 77% of the control group. The main reasons for early termination in both groups included: withdrawn consent (25 vs. 50%), adverse events (38 vs. 33%), lost to follow-up (13 vs. 17%) or other (25 vs. 0%). The baseline characteristics of the ITT population are shown in Table 2. There was no statistically significant difference in baseline characteristics between groups. The number of subjects with possible risk factors (e.g., smoking, diabetes, osteoporosis) was low. The mean skin thickness as measured during surgery (prior to incising the skin), was similar in both groups.

**Table 2:** Subject demographics of the ITT population.

	**Test group BIA400 system (*n* = 51)**	**Control group BIA300 system (*n* = 52)**
Gender, *n* (%)		
Male	26 (51.0%)	25 (48.1%)
Female	25 (49.0%)	27 (51.9%)
Age, mean (SD)	54.2 (10.9)	51.5 (16.6)
Type of hearing loss, n (%)		
Conductive	5 (9.8%)	10 (19.2%)
Mixed	31 (60.8%)	35 (67.3%)
SSD	15 (29.4%)	7 (13.5%)
Subjects per country and site, *n* (%)		
The Netherlands (total)	26 (51.0%)	29 (55.8%)
Maastricht	14 (27.5%)	15 (28.8%)
Breda	6 (11.8%)	8 (15.4%)
Deventer	4 (7.8%)	3 (5.8%)
Eindhoven	2 (3.9%)	3 (5.8%)
Spain		
Valencia	15 (29.4%)	14 (26.9%)
France		
Toulouse	5 (9.8%)	5 (9.6%)
Sweden		
Göteborg	5 (9.8%)	4 (7.7%)
Nicotine usage, *n* (%)	14 (27.5%)	13 (25.5%)
Relevant medical history, *n* (%)		
Diabetes	3 (5.9%)	2 (3.8%)
Osteoporosis^*^	1 (2.0%)	1 (1.9%)
Skin thickness in mm, mean (SD)	6.3 (1.58)	6.2 (1.71)

*SSD single-sided sensorineural deafness*.

* *Patients who had severe osteoporosis as judged by the investigator were to be excluded*.

### Summary of Relevant Protocol Deviations, Data Cleaning, and Missing Values

The number of missing values for the primary efficacy outcome measure was 3.0% for the test and 4.0% for the control group during the first year, and 0.8 and 1.4% for the last two years. Protocol deviations were dominated by deviations from the stipulated time visit windows (*n* = 55), missed visits (*n* = 17), delayed sound processor loading (n = 25) and procedural errors in data collection, namely scale reversals, scale misinterpretations and providing wrong instructions to the subject (*n* = 18). Other relevant deviations included leaving a rotationally mobile implant in place during surgery (subject excluded from the PP population, but included in the ITT population), a deviation in the type of skin incision during surgery and erroneous allocation of abutment type after randomization (subject withdrawn from study following surgery, hence only included in Safety population) (Figure 2).

In relation to the *Clean file* meeting, prior to data database lock and data analysis, some relevant clarifications of clinical data were necessary. Relevant modifications included three instances of a Holgers 4 assessment, which were rescaled to Holgers 3, as the abutment was not removed by the clinician. The latter was a predefined requirement, as prescribed in the protocol. For one subject, a Soft tissue thickening/overgrowth scale entry conflicted with both the visible abutment length measurement and adverse event page. This value was replaced by the (higher) value which was entered in the adverse event page. Two subjects were wrongly instructed in completing the POSAS scale which lead to a reversal of scale, the two evident outliers were removed from the ITT dataset.

### Primary Efficacy Analysis

**Figure 3:**
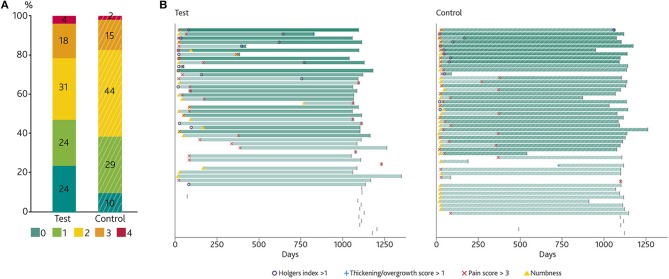
The primary combined endpoints. **(A)** Stacked bar chart showing the percentage of subjects presenting with 0, 1, 2, 3, or 4 of the four important medical events (Holgers Index > 1, soft tissue thickening/overgrowth > 1, Pain > 2 or the presence of numbness) comprised in the combined primary variable at any point over the first year and until the end of the study. Every event is counted only once per subject. **(B)** Time of onset of these events presented per patient, which enables an individual and group analyses over time. The stepwise increase in intensity (in green) illustrates an incremental amount of experienced medical events (a blank bar representing 0 events). The graph does not show the duration of the events. The last day of data collection in the study for the first year is indicated by a vertical line.

Analysis of the combined primary efficacy variable (numbness, pain, peri-abutment dermatitis and soft tissue thickening/overgrowth) at 1 year showed a difference between the two groups (Figure 3) which did not reach statistical significance for the ITT population (*p* = 0.12). The difference did reach statistical significance in the PP population (*p* = 0.033) in favor of the test group. An analysis, including only the subjects in the PP population who attended all study visits (*n* = 90) yielded a comparable *p*-value as the ITT analysis (*p* = 0.14). As shown in Figure 3 (ITT analysis), more subjects in the test group (29%) experienced none of the four medical events comprised in the combined variable, compared to the control group (13%). There was no statistically significant difference between the two groups after 3 years (*p* = 0.45). The amount of subjects who had no relevant medical events decreased to 24% and 10% for the test and control group, respectively. Figure 3B shows the time point at which each subject experienced any of the four events for the first time; the figure shows that numbness occurred mostly after surgery (primarily in the control group) and was sometimes followed by the onset of pain.

### Secondary Efficacy Analyses

Secondary efficacy outcomes are presented in Table 3. As the results for the ITT and PP populations were similar, only results for the ITT population are presented. Results that showed a statistically significant difference are presented. The surgical time is statistically significantly different (*p* < 0.001) between treatment groups with a mean surgery time for the test group of 15.3 min (CI 13.6–17.1) and 24.7 min (CI 22.3–27.1) min for the control group. The skin at the surgical site was reported to be healed in 92% of the test subjects at the first post-operative visit 10 days after surgery, compared to 73.1% of the control subjects (*p* = 0.020). The corresponding percentages were 92.0 vs. 84.6% (*p* = 0.40) 3 weeks post-surgery, and 97.9 vs. 100% (*p* = 0.97) at 3 months. The median time to sound processor loading for the test group was 3.7 weeks (min-max 1.9–12.7 weeks) and 4.0 weeks (min-max 2.6–15.0 weeks) for the control group.

**Table 3:** Outcomes in the ITT population.

	**Test group (*n* = 51)**	**Control group (*n* = 52)**	***P*-value**
Abutment length, *n* (%)			NP
6 mm	2 (3.9%)	47 (90.4%)	
8 mm	15 (29.4%)	N/A	
9 mm	N/A	5 (9.6%)	
10 mm	22 (43.1%)	N/A	
12 mm	12 (23.5%)	N/A	
Surgical time, minutes (mean, SD, 95% CI, min; max)	15.3 (6.2) (13.6; 17.1) (7.0; 32.0)	24.7 (8.6) (22.3; 27.1) (10.0; 45.0)	<.0001^*^
Wound healed before visit 1, *n* (%)	47 (92.2%)	38 (73.1%)	0.020^*^
Time to sound processor loading, weeks (mean, SD, median, min; max)	5.31 (3.21) 3.71 (1.86; 12.71)	5.57 (3.31) 4.00 (2.57; 15.00)	0.66
Maximum of pain (up to 3 years) Neuropathic pain, *n* (%)			0.076
No	34 (66.7%)	27 (51.9%)	
Mild	7 (13.7%)	8 (15.4%)	
Moderate	7 (13.7%)	9 (17.3%)	
Severe	3 (5.9%)	8 (15.4%)	
Direct pain, *n* (%)			0.71
No	9 (17.6%)	9 (17.3%)	
Mild	20 (39.2%)	14 (26.9%)	
Moderate	13 (25.5%)	23 (44.2%)	
Severe	9 (17.6%)	6 (11.5%)	

*NP not performed. N/A not available*.

* *Denotes statistical significance (P < 0.05)*.

**Figure 4:**
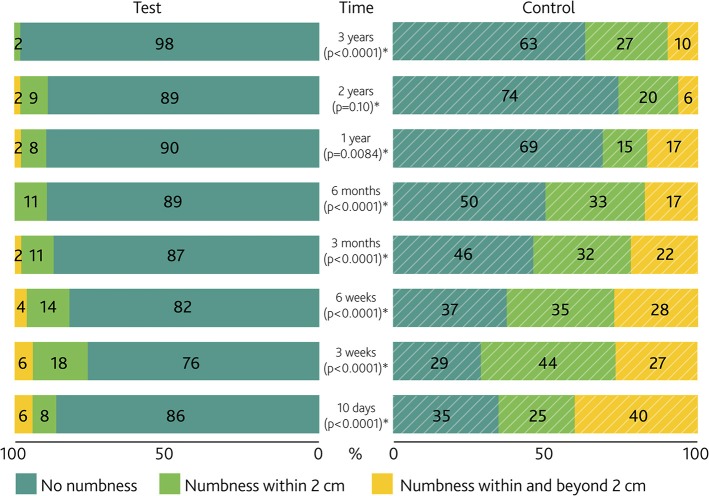
Numbness severity per visit. Paired bar chart of the percentage of numbness around the abutment in the test and control group per study visit. *P*-values for the difference between groups are presented. *Denotes a significance level of *p* < 0.05.

As shown in Figure 4, statistically significantly less numbness was recorded in the test group at all time points throughout the study period, except for the 2 year visit (*p* = 0.10). At 3 years, 89.1% of the test group was free of any numbness in comparison to 63% in the control group.

**Figure 5:**
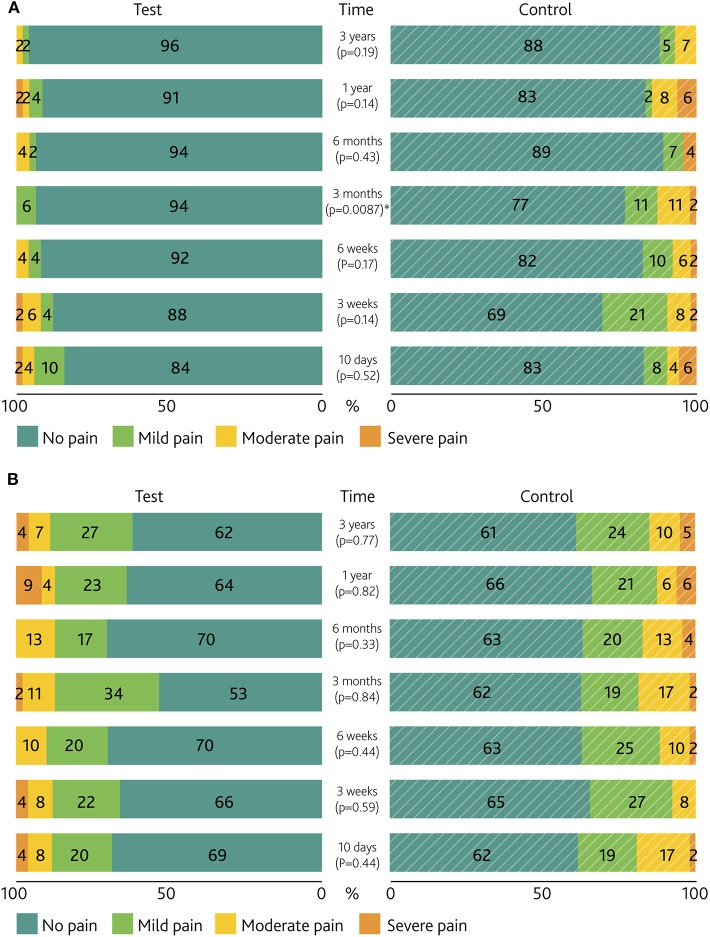
Pain severity per visit. Paired bar chart of the percentages of **(A)** *neuropathic pain* and **(B)** *direct pain* surrounding the abutment and/or scar. *P*-values for the difference between groups are presented. *Denotes a significance level of *p* < 0.05.

For the neuropathic dimension of the pain score, categorized as No, Mild, Moderate or Severe pain (Figure 5A), a statistically significant difference was seen at 3 months (*p* = 0.0087). The maximum experienced neuropathic pain during the first year per subject (Table 3) shows a trend in favor of the test group (*p* = 0.076), which remained unchanged until the end of the study. No statistically significant differences existed in the occurrence of direct pain (Figure 5B).

**Figure 6:**
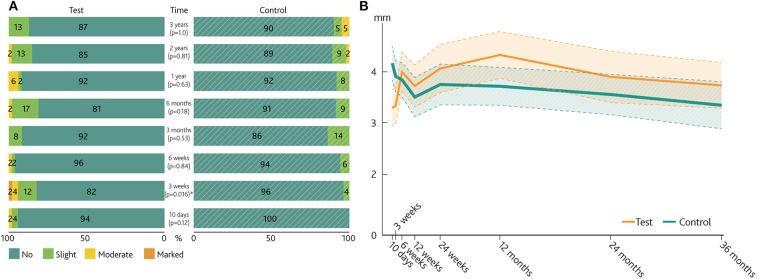
Soft tissue thickening/overgrowth and visible abutment length. **(A)** Paired bar chart of the Soft tissue thickening/overgrowth scale of the test group and control group. *P*-values for the difference between groups are presented. **(B)** Mean visible abutment length over time (linearly interpolated between visits). Dashed lines represent the 95% confidence intervals for the mean. *Denotes a significance level of *p* < 0.05.

The analysis of soft tissue thickening/overgrowth scores (Figure 6) showed low values for the majority of subjects in both groups, with a few instances of moderate/marked soft tissue thickening being reported in the test group.

A statistically significant difference was noted at 3 weeks, with more swelling reported for the test group (*p* = 0.016). After 3 years, 87% of the test group had no soft tissue thickening or overgrowth vs. 90% in the control group. In contrast, five abutments had to be exchanged to longer abutments due to overgrowth in the control group compared to four abutments in the test group. The measurements of visible abutment length showed relatively lower values at the earliest time point following surgery for the test abutment compared to measurements performed at later time points. The opposite trend was noted for control abutments, where larger visible abutment lengths recorded at the early time points were followed by lower values at subsequent visits.

**Figure 7:**
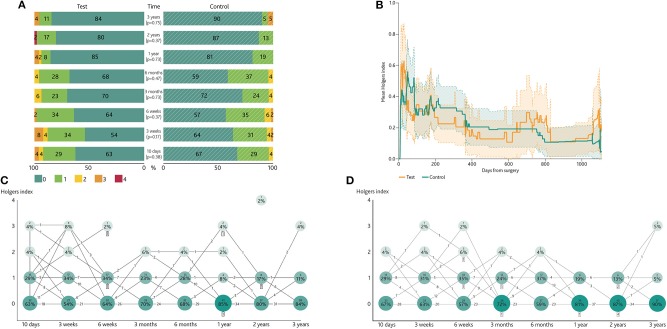
Peri-abutment dermatitis measurements. **(A)** Paired bar chart of the percentages of the Holgers index per scheduled visit. **(B)** Mean Holgers score over time recorded at scheduled study visits and unscheduled extra visits (adverse event). As there are more subjects with a Holgers Index of 0 at any point in time, the mean Holgers Index is lower than the lowest state of inflammation (Holgers Index of 1). Dashed lines indicate 95% confidence intervals for the mean. **(C)** Changes in Holgers index between scheduled visits for the test group. **(D)** Changes in Holgers index between scheduled visits for the control group. The numbers in the circles show the percentage and number of subjects per Holgers state at each time point. The numbers on the lines describe the numbers of subjects that undergo a state change between time points. The number in the squares represents the number of subjects that are a lost to follow up. Some connections do not sum due to missing data.

No statistically significant differences in peri-abutment dermatitis as evaluated by Holgers scores were recorded at any of the pre-defined study visits (Figure 7A), when analyzing the mean over the first year (test 0.33 ± 0.41 vs. control 0.34 ± 0.33, *p* = 0.51) or over the total study duration (test 0.27 ± 0.41 vs. control 0.24 ± 0.28, *p* = 0.62).

The time of occurrence of peri-abutment dermatitis was noted when also considering Holgers scores reported at unscheduled extra visits (Figure 7B). For this unconventional way of using the Holgers Index, this index was averaged per subject, per day. This led to a score which represents the burden of peri-abutment dermatitis for the whole treatment group. The trend indicated a gradual stabilization in peri-abutment dermatitis over time for both groups. Figures 7C,D display the changes in Holgers Index per visit per subject for both treatment groups. This enables an assessment of how peri-abutment dermatitis develops over time in clusters of subjects (e.g., assess how likely it is to go from a Holgers 0 at 3 weeks to Holgers 3 at 6 weeks for someone in the test group). The maximum Holgers index shows that over the course of the study, 17.6% of the subjects in the test group were free of any inflammation (Holgers 0) vs. 15.4% in the control group. A moderate to severe inflammation (Holgers 2-4) occurred in 37.2% of the test group vs. 25% in the control group during that time. These categorized maximum score differences (including Holgers 1) were not statistically significant between groups (*p* = 0.12).

Esthetic outcomes, as measured using the POSAS scale, are shown in Table 4. In general, the mean overall opinion scores were low for both groups (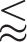
3). At 3 months, statistically significant differences existed as measured by the investigator (observer) for most individual attributes of the POSAS scale and for the overall opinion in favor of the test group (*p* = 0.002). Ratings by the subject, however, did not reach statistical significance (*p* = 0.16). At 1 year, the difference in ratings (Overall opinion) between the groups by the observer diminished. A statistically significant difference for the observer existed (total score, test 10.9 ± 5.6 vs. control 14.8 ± 8.9, *p* = 0.03). A subset of qualitative items relating to the difference in pliability, surface and/or stiffness of the skin around the abutment assessed by the observer or subject also achieved statistical significance in favor of the test group.

**Table 4:** Esthetic outcomes as measured with POSAS in the ITT population.

	**Test group (*n* = 51)**		**Control group (*n =* 52)**		***P*-value (between groups)**	
	**Observer**	**Patient**	**Observer**	**Patient**	**Observer**	**Patient**
**POSAS**^†^ **(Mean, SD) at 3 months**						
Vascularity & Pigmentation | Color	2.11 (1.15) 1.96(1.16)	2.32(1.49)	2.81 (1.54) 2.21(1.27)	2.06(1.29)	0.03^*^ 0.30	0.41
Thickness	2.26 (1.71)	2.35 (1.89)	3.25 (1.49)	2.60 (1.64)	<0.001^*^	0.13
Relief & Surface area | Irregular	2.11 (1.18) 2.04(1.12)	2.89(2.14)	3.15 (1.56) 3.06(1.34)	3.27(2.07)	<0.001^*^ <0.001^*^	0.18
Pliability | Stiffness	2.21 (1.10)	2.30 (1.71)	3.23 (1.75)		<0.002^*^	0.43
Pain not within the scar area		1.00 (0.00)		1.62 (1.50)		0.002^*^
Total score	12.7 (5.8)	14.3 (7.6)	17.7 (7.4)	15.1 (6.5)	<0.001^*^	0.28
Overall opinion	2.28 (1.24)	2.33 (1.69)	3.02 (1.22)	*2.81 (2.13)*	0.002^*^	*0.16*
**POSAS**^†^ **change from 3 months to 1 year (Mean, SD)**						
Overall opinion	−0.24 (1.65)	−0.56 (1.62)	−0.36 (1.87)	−0.64 (2.37)	0.49	0.94
*P*-value within group	0.15	0.02^*^	0.16	0.15		
**POSAS**^†^ **(Mean, SD) at 1 year**						
Vascularity & Pigmentation |Color	1.98 (1.34) 1.69(1.26)	1.78(1.73)	2.28 (1.48) 1.91(1.21)	1.52(1.24)	0.33 0.23	0.98
Thickness	2.02 (1.52)	1.80 (1.85)	2.72 (1.99)	1.89 (1.62)	0.06	0.33
Relief & Surface area | Irregular	1.96 (1.24) 1.94(1.28)	1.87(1.78)	2.74 (1.87) 2.68(1.70)	2.00(1.68)	0.08 0.02^*^	0.38
Pliability | Stiffness	2.08 (1.22)	1.76 (1.75)	2.66 (1.70)	1.87 (1.57)	0.14	0.25
Pain not within the scar area		1.09 (0.58)		1.67 (1.92)		0.05
Total Score	11.7 (6.9)	11.7 (9.5)	15.0 (9.0)	11.9 (8.1)	0.09	0.44
Overall opinion	2.00 (1.29)	1.80 (1.31)	2.60 (1.65)	2.15 (1.53)	0.08	0.23
**POSAS**^†^ **change from 3 months to 3 years (Mean, SD)**						
Total Score	−1.84 (6.59)	−2.12 (10.56)	−2.37 (9.65)	−1.91 (8.73)	0.69	0.66
P value within group	0.067	0.012^*^	0.029^*^	0.43	-	-
Overall opinion	−0.30 (1.47)	−0.41 (2.03)	−0.50 (1.59)	−0.45 (2.77)	0.21	0.98
P value within group	0.20	0.11	0.032^*^	0.31	-	-
**POSAS**^†^ **(Mean, SD) at 3 years**						
Vascularity & Pigmentation |Color	1.69 (1.33) 1.62(1.01)	1.80(1.96)	2.13 (1.49) 2.03(1.61)	1.58(1.63)	0.09 0.48	0.76
Thickness	2.02 (1.31)	2.09 (1.96)	2.63 (1.85)	2.13 (1.68)	0.11	0.65
Relief & Surface area | Irregular	1.93 (1.18) 1.98(1.11)	1.89(1.90)	2.80 (1.86) 2.68(1.62)	2.35(1.97)	0.03^*^ 0.045^*^	0.08
Pliability | Stiffness	1.69 (1.06)	1.62 (1.61)	2.58 (1.60)	2.05 (1.60)	0.001^*^	0.02^*^
Pain not within the scar area		1.000 (0.00)		1.27 (1.03)		0.07
Total score	10.9 (5.6)	11.8 (10.1)	14.8 (8.9)	12.6 (8.7)	0.03^*^	0.19
Overall opinion	1.93 (1.05)	1.82 (1.65)	2.43 (1.52)	2.23 (1.79)	0.15	0.08

† *A selection of quantitative measures. Attributes were tabulated together (| and &) to reflect similar entities across the observer (left side) and patient (right side) scores*.

* *Denotes statistical significance (P < 0.05)*.

### Adverse Events

Reported adverse events are summarized in Table 5. A total of 223 adverse events that were probably or definitely related to the intervention were recorded throughout the study. These adverse events were present in 56% of the subjects in the test group and 52% of the control group in the safety population. In general, no large differences existed in the incidence or type of adverse events between the two groups. Dehiscence of the skin in the area around the abutment was present in 1.9% of the test group and 11.5% of the control group during the first year. Two implants were lost during the duration of the study, one in each group, resulting in a cumulative implant survival rate of 97.9% and 96.2% in the test and control group of the safety population, respectively. The lost test implant did not reach rotational stability at the time of implantation, and remained rotationally mobile at subsequent visits. One control implant was lost after a period of peri-abutment dermatitis in an otherwise healthy middle-aged subject and no likely causal relation could be established. Both implants were lost 52 days after surgery. In addition, one implant was removed in the control group due to ongoing pain after the abutment was removed. Four abutments were removed throughout the study, two in the test group and two in the control group. In the test group one abutment was removed due to Holgers 4 inflammation and one was removed due to Holgers 3 inflammation and overgrowth. In the control group both abutments were removed due to ongoing pain, one of these led to subsequent removal of the implant. The two implant losses and the removed abutment were the only device-related events that were classified as serious adverse events.

**Table 5:** Adverse events in the safety population.

	**Test group** **(*n* = 53)**	**Control group** **(*n* = 53)**	***P*-value**
Subjects with one or more AEs, *n* (%)			
AE	35 (67.3%)	39 (75.0%)	0.52
Related^†^ AE	29 (55.8%)	27 (51.9%)	0.84
Serious AE	7 (13.5%)	10 (19.2%)	0.60
Related^†^ Serious AE	1 (1.9%)	3 (5.8%)	0.62
Subjects with relevant AEs, *n* (%)			
Implant extrusion	1 (1.9%)	2 (3.8%)	NP
Abutment removal	2 (3.8%)	2 (3.8%)	NP
Dehiscence	1 (1.9%)	6 (11.5%)	NP

† Adverse events (AE) which are probably or definitely related to the treatment. A selection of relevant AEs for which no strict outcome measure was defined. NP not performed.^*^Denotes statistical significance (P < 0.05).

Pain scores are presented in Figure 5.

## Discussion

### Summary and Interpretations of Findings

The primary aim of this study was to assess the difference in complications between two different abutments and surgical techniques for bone conduction hearing implants: a HA-coated abutment placed using soft tissue preservation surgery in comparison with a titanium abutment placed using a soft tissue reduction technique. The simultaneous change of surgical method and abutment type hampers comparison of attributes, thus making it a pragmatic trial, reflecting clinical practice. The difference between the two treatment groups in terms of the combined primary variable (peri-abutment dermatitis, soft tissue thickening/overgrowth, pain and numbness) did not reach statistical significance for the ITT population after 1 year (ITT *p* = 0.12, PP *p* = 0.03) or three years (ITT *p* = 0.45). Over twice as many subjects in the test group (ITT) had an uneventful first year in terms of these complications. The largest group of subjects in the control group (46%) had at least two important medical events whereas in the test group the largest groups consisted of subjects who had zero or up to two events (Figure 3). This effect was smaller at 3-year follow-up (24 vs. 10%).

When analyzing each of the parameters contained within the combined variable separately, several parameters achieved statistical significance when comparing interventions. A significant difference existed in terms of numbness around the abutment. A certain prevalence of numbness can be expected as a result of surgery, but in the test group, 86% of subjects were already free of numbness at the first post-operative visit and 90% were free of numbness after 1 year. In the control group, the percentage of subjects without numbness increased from 35% to 69% during the same period. These proportions remained stable over the last 2 years. As sensibility can be related to the perception of bodily integrity (41), subjects may be reminded daily of a loss of sensibility upon coupling the sound processor to the abutment. No large differences existed in direct pain surrounding the abutment between the groups; however, direct pain led to removal of one test and one control abutment during the study. The difference in neuropathic pain between the two groups may be partially attributed to less nerve damage and enhanced nerve repair in the preserved, full-thickness skin in the test group. Neuropathic pain (42) is known to affect quality of life, it is clinically often resistant to treatment and can lead to elective abutment or implant removal (43), which was the case for one subject in the control group in this investigation. During the last 2 years of the study there was no increase of maximum neuropathic pain in any subject, whereas five subjects in both groups experienced a higher maximum direct pain. Nevertheless, the cause and prevention of both neuropathic and direct pain remain a point of attention for the future as it was still a relatively common finding in both groups at the end of the first year.

There was significantly more soft tissue thickening in the test group during the initial period post-surgery. This may be explained by the preserved ability of full-thickness skin to swell in reaction to surgical trauma and inflammation. From 6 weeks after surgery until the end of the study, no statistically significant difference in the occurrence of soft tissue thickening could be seen between the two groups. The measurements of visible abutment length suggested that the average skin level regressed in the test group during the first year, while in the control group the skin thickened. At 3-year follow-up, the visible abutment length was 0.3 mm longer for the test group, which featured longer abutments. Of clinical importance, and in contradiction with the recorded soft tissue thickening scores, five abutments had to be replaced in response to skin overgrowth in the control group, compared to just one in the test group during the first year. After 3 years, the total number of exchanged abutments due to overgrowth was still five in the control group, while the number had increased to three in the test group. The discrepancy between soft tissue thickening scores and number of abutment removals during the first year raises concerns regarding the validity of the newly introduced soft tissue thickening/overgrowth scale. The results suggest that the scale might not be suitable for comparing preserved vs. unpreserved skin around the abutment in terms of soft tissue swelling. The Holgers index is also not sensitive to this type of soft tissue reaction.

In general, no statistically significant differences existed between the two study interventions in terms of peri-abutment dermatitis (Holgers index) alone. The non-statistically significant trend in Holgers Index, together with the results from the measurements of soft tissue thickening and visible abutment length suggest that the test group experienced more inflammation than the control group at short-term follow up. From a pathophysiological perspective, this may be explained by the presence of intact subcutaneous tissue in the test group, which preserves its natural capacity to produce exudate, swell or increase local blood flow (44), in contrast with the control group where the natural healing capacity was hampered by the absence of subcutaneous tissues. Furthermore, skin movements around the abutment may cause shear stresses that can lead to peri-abutment dermatitis. The data suggests that the preserved skin in the test group is influenced more strongly by these factors during the post-surgical period. The process of skin integration to the HA coating of the abutment might also play a role. Increased inflammation has previously been observed in a non-randomized retrospective comparison between an all-titanium and HA-coated abutment (45), but was suggested to be related to the sound processor loading time.

An important concern is whether the current Holgers index, which was initially designed and used to evaluate the implant site following soft tissue reduction surgery, is suitable for evaluating full thickness skin around an abutment. Recently, there have been proposals to use a different peri-abutment dermatitis scale (46–48). However, these scales lack validation and their reproducibility is unknown. In general, more than 80% of subjects experienced at least one period of minor inflammation. Up to 37% of subjects experienced a moderate or severe period of peri-abutment dermatitis. The overall high incidence of inflammation reported calls for more interventional research specifically aimed at reducing peri-abutment dermatitis. A reduction of the incidence of peri-abutment dermatitis was not achieved with the test intervention. A significant reduction of 38% in mean surgery time was achieved for the test group by omitting soft tissue reduction. Wound healing after surgery was significantly faster in the test group, which can be explained by a less invasive approach and the preservation of subepidermal structures that play an important role in the wound healing process (44). Based on previous studies, it is also plausible that properties of the HA-coating may have aided wound healing as it is able to adsorb proteins and host cells of the skin (32), which may promote the establishment of a seal between soft tissue and the abutment. From this trial, the attributes to the surgical and abutment factors to this improved outcomes cannot be established.

The cosmetic ratings by the non-blinded observers, as measured with the POSAS scale, revealed a significant difference in total score between the two groups during short and long-term follow-up. The subjects' own rating yielded no significant differences between groups, except for a qualitative rating of stiffness after 3 years in favor of the test intervention. No large differences were to be expected as subjects have no reference value, the area is not easily assessed by the subjects themselves, and the attributes do not directly relate to where the main differences between two interventions lie (e.g., baldness). This may also explain the relative improvement in POSAS outcomes compared to the observer who can, to a lesser extent, also be expected to be affected by the lack of blinding.

The general intervention-related adverse events were equally distributed. Although implant losses are burdensome for patients, an overall implant loss incidence of 2% was reported during the first 3 months. No further implants were lost or extruded during the remainder of the study, which demonstrates that secondary infections play a minor role in implant survival.

### Future Developments

There was a general improvement in outcomes during the 3 years for the test group. Delayed complications did exist, such as skin inflammation at 3 years [e.g., Holgers index 3 occurrence of 4% (test) vs. 5% (control)]. The skin-abutment interface remains intrinsically prone to adverse reactions and clinical investigations should, try to adapt the scales (45) to be relevant for full-thickness skin and to validate the outcome measures.

### Limitations

Two variables have been tested in this trial. Prior to the advent of this clinical trial, a consensus was published that soft tissue reduction was important to achieve acceptable soft tissue complication rates with a smooth titanium abutment (16). This led to the development of the current HA-coating to stabilize the full-thickness soft tissue surrounding the abutment. This study did therefore not address soft tissue preservation with an all-titanium abutment and it cannot be directly investigated to what extent the results are separately influenced by the soft tissue preservation or the HA-coating. Instead, this pragmatic clinical trial resembles clinical practice when the new abutment was introduced. Favorable results using an all-titanium abutment and soft tissue preservation surgery have now also been shown in a smaller, but well-designed study (49). Additionally, a review also highlighted that tissue preservation during surgery may reduce postoperative skin complication rates (50). While this is the largest prospective randomized clinical trial in bone conduction hearing implant research to be reported, detectable effect sizes remain relatively small in comparing peri-abutment dermatitis between groups in an international multi-center setting. Reliable and validated (primary) outcome measures in small trials are thus important and warrant further investigation. Also, the weight of the different types of complications which are included in the primary outcome measure should be further investigated. The duration of follow-up is expected to capture the incidence of common complications, such as moderate to severe peri-abutment dermatitis, encountered by subjects (50, 51). No adjustments for multiple testing were carried out, but general effects observed were consistent in their direction over time. The outcomes might be different for the two interventions in the pediatric population.

## Conclusion

Soft tissue preservation with a HA-coated abutment leads to a reduction in the combined occurrence of complications over the first year which is not statistically significant in the ITT population but is in the PP population. This effect decreased for the long term study follow up of 3 years and did also not reach statistical significance. The improvement in the secondary efficacy outcome measures such as numbness, surgical time and the cosmetic results did reach statistical significance on several time points and can be considered clinically meaningful in comparison to soft tissue reduction surgery with an all-titanium abutment. The results demonstrate that the new, combined treatment modality is effective and safe over the short and long term.

## Data Availability Statement

The datasets generated for this study are available on request to the corresponding author.

## Ethics Statement

The studies involving human participants were reviewed and approved by METC MUMC+. The patients/participants provided their written informed consent to participate in this study.

## Author Contributions

Design: MH, SW, JB, MJ, JS, ME-O, MF, and RS. Conduct: MH, DM, SB, JS, ME-O, OD, AR, JA, and RS. Analyses and manuscript drafting: MH, SW, JB, MJ, and RS. Editing, review, and approval: All authors.

### Conflict of Interest

This research was sponsored and funded by Cochlear Bone Anchored Solutions AB in full. SW, MF, and JB were paid employees of Cochlear Bone Anchored Solutions AB. MH declares a travel grant of Cochlear Bone Anchored Solutions AB. MM and HA are paid consultants to Cochlear Bone Anchored Solutions AB. The remaining authors declare that the research was conducted in the absence of any commercial or financial relationships that could be construed as a potential conflict of interest.
