# Supplementary material for: Clinical Outcomes of Soft Tissue Preservation Surgery With Hydroxyapatite-Coated Abutments Compared to Traditional Percutaneous Bone Conduction Hearing Implant Surgery—A Pragmatic Multi-Center Randomized Controlled Trial
Source: Front Surg. 2020 Mar 5;7:5. doi: 10.3389/fsurg.2020.00005 (PMC7066494; doi:10.3389/fsurg.2020.00005)
Supplement: Supplementary file 1 [file Data_Sheet_1.docx]

**Supplementary appendix**

**Full list of authors**

M. van Hoof, S. Wigren, J. Ivarsson Blechert, M.A. Joore, M. Molin, J.R. Hof, J. van Tongeren, J.W. Brunings, L.J.C. Anteunis, M. I. Rincon Piedrahita, M.P. Martinez-Beneyto, D.J.M. Mateijsen, S.J.H. Bom, J. Stalfors, M. Eeg-Olofsson, O. Deguine, A.J.M. van der Rijt, M.C. Flynn, J. Marco Algarra, R.J. Stokroos

| Procedures and timing | Visit 1  Baseline | Visit 2 Surgery | Visit 3 | Visit 4 | Visit 5 | Visit 6 | Visit 7 | Visit 8 | Visit 9 | Visit 10 |
| --- | --- | --- | --- | --- | --- | --- | --- | --- | --- | --- |
| Day/Week/Month | Before day of surgery | D 0 | D 10 | W 3 | W 6 | W 12 | W 24 | M 12 | M 24 | M 36 |
|  | | | | | | | | | | |
| Demographics | X |  |  |  |  |  |  |  |  |  |
| Informed consent | X |  |  |  |  |  |  |  |  |  |
| Randomisation^†^ |  | X |  |  |  |  |  |  |  |  |
| Skin thickness |  | X |  |  |  |  |  |  |  |  |
| Length of abutment |  | X |  |  |  |  |  |  |  |  |
| Implant surgery |  | X |  |  |  |  |  |  |  |  |
| Implant stability |  | X | X | X | X | X | X | X | X | X |
| Suture removal |  |  | X |  |  |  |  |  |  |  |
| Wound healing |  |  | X | X | X | X | X |  |  |  |
| Sound processor installation |  |  |  | X |  |  |  |  |  |  |
| Daily use of sound processor |  |  |  |  | X | X | X | X | X | X |
| Change of abutment |  |  | X | X | X | X | X | X | X | X |
| Loss of implant |  |  | X | X | X | X | X | X | X | X |
| Holgers index |  |  | X | X | X | X | X | X | X | X |
| Soft tissue thickening/overgrowth |  |  | X | X | X | X | X | X | X | X |
| Visible abutment length |  |  | X | X | X | X | X | X | X | X |
| Esthetic evaluation surgeon |  |  |  |  |  | X |  | X |  | X |
| Esthetic evaluation subject |  |  |  |  |  | X |  | X |  | X |
| Pain |  |  | X | X | X |  | X |  |  | X |
| Numbness |  |  | X | X | X | X | X | X | X | X |
| Health Utility Index | X |  |  |  |  |  | X | X |  | X |
| Abbreviated Profile of Hearing Aid Benefit | X |  |  |  |  |  | X | X |  | X |
| Extra visits |  |  | X | X | X | X | X | X |  |  |
| Concomitant treatment |  |  | X | X | X | X | X | X |  |  |
| Concomitant medication |  |  | X | X | X | X | X | X |  |  |
| Device deficiency |  | X | X | X | X | X | X | X | X | X |
| Adverse events |  | X | X | X | X | X | X | X |  |  |

**S1 Supplemental Figure 1. Trial flow chart**

The trial flow chart of the full investigation (3 years) for all relevant procedures and outcome measures. ^†^Randomization was performed between the baseline and surgery visit.
